# Dominance of ascomycota and ubiquity of *Trichoderma* in *Argemone mexicana* microbiomes across Assam, India

**DOI:** 10.3389/ffunb.2026.1868321

**Published:** 2026-10-08

**Authors:** Pooja Singh, Angkita Sharma, Bidyut Kumar Sarmah, Preeti Arivaradarajan, Tankeswar Nath, Shoma Paul Nandi

**Affiliations:** 1 Amity Institute of Biotechnology, Amity University, Noida, Uttar Pradesh, India; 2 DBT-AAU Centre, Assam Agricultural University, Jorhat, Assam, India; 3 Department of Microbiology, Thiagarajar College, Madurai, Tamil Nadu, India

**Keywords:** *Argemone mexicana*, ascomycota, fungal community diversity, metagenomic analysis, rhizospheric soil, root, shoot, *Trichoderma* species

## Abstract

**Introduction:**

In India, *Argemone mexicana* is known for its pharmaceutical value. The plant hosts many endophytes. Plant-associated microbes also help plants with development and protection.

**Material and methods:**

A metagenomic study of rhizospheric soil, roots, and shoots was conducted to investigate variation in the organization and diversity of fungal communities from two different locations (Amlighat and Dergaon, Assam, India) for the first time. Illumina sequencing was used for the fungal metagenomic analysis. The DNA library was constructed using primers specific for the fungal internal transcribed spacer 2 (ITS2) region.

**Results and discussion:**

Next-generation sequencing identified a total of 5 phyla, 32 classes, 39 orders, 68 families, 125 genera, and 273 fungal species (including unknown and unidentified taxa) in triplicate samples. The fungal taxa were mostly similar in both locations but occurred at varying relative abundances. The fungal community richness was higher in the root samples from Dergaon than in the other samples. Ascomycota (37.47%–75.84%) was the dominant phylum across all samples, followed by Basidiomycota (3.53%–6.95%). Beta diversity analyses, including principal component analysis, cluster analysis, unweighted pair group method with arithmetic mean (UPGMA), and network analysis, also showed a close relationship between the fungi of both locations. We observed mostly similar fungal diversity, with some differences, among the roots and shoots of *A. mexicana* and its rhizospheric soil. *Trichoderma* species were detected in all samples at varying relative abundances. These findings suggest that this fungus may modulate acaricide production in *A. mexicana*.

## 1 Introduction

The advancement in omic techniques has paved the way for microbial diversity studies (Brader et al., 2017; Alawiye and Babalola, 2019). To date, nearly 5% of the actual (estimated) number of microbial species have been recognized. This limitation has been mitigated through metagenomic studies, which have assisted us in understanding the diversity of microbes in different habitats. These studies have included microbes from various locations such as the upper phyllosphere, rhizosphere, and plant tissues (Bulgarelli et al., 2013; Strobel and Daisy, 2003; Strobel, 2003; Toju et al., 2018). The current study focuses on using metagenomic next-generation sequencing (mNGS) to compare culturable and non-culturable fungal endophytes. The mNGS technology is used for fungal/bacterial marker-gene amplicon sequencing and can characterize microbial communities in any sample (rhizosphere, phyllosphere, and inside the plant) (Shurigin et al., 2026; Alharbi et al., 2025; Liu et al., 2025; Yang et al., 2022; Strobel and Daisy, 2003).

The current study focuses on endophytic (root and shoot) and rhizospheric fungal communities from the *A. mexicana* plant. *A. mexicana* (order: Papaverales; family: Papaveraceae) is an exotic weed and is indigenous to South America and is distributed in various tropical and subtropical countries. *A. mexicana* is a very important plant in India with immense medicinal properties. It has an important role in the pharmaceutical industry as it contains some medicinally important secondary metabolites (Kulshrestha et al., 2026; Omsatyam et al., 2026; Konika et al., 2026; Saraswat et al., 2025; Bhadra and Vasundhara, 2025; Patocka et al., 2024; Bhadra and Vasundhara, 2024; De la Cruz-Velueta et al., 2024; Jaiswal et al., 2023). Some important compounds of *A. mexicana* are berberine, protopine, sanguinarine, jatrorrhizine, and columbamine. Protopine is used as an analgesic compound. Berberine has many medicinal properties, including antimicrobial, antiprotozoal, antidiabetic, anti-inflammatory, antioxidant, antiautophagic, and anticancer activities at prescribed doses (Patocka et al., 2024). Lou et al. (2021) reported that sanguinarine showed anticancer effects through inhibition of invasion, proliferation, and angiogenesis and induction of apoptosis in cancer lesions (Lou et al., 2021). Jatrorrhizine shows antimicrobial, anticancer, hypolipidemic, anti-obesity, anti-diabetic, and antiprotozoal activities and other valuable properties, such as effects on the central nervous system (Zhong et al., 2022). Many other secondary metabolites, including columbamine, are used in medicine. Berberine and sanguinarine are also used as acaricidal agents (tick killers) (Upadhaya et al., 2020).

The host plants and the associated endophytes produce similar secondary metabolites (Strobel, 2003). Information regarding the total fungal composition may help us understand the patterns of plant microbial colonization and the contribution of these microbes to metabolite production (Singh and Kumar, 2023). Thus, we used a next-generation sequencing (NGS) approach to characterize the culturable and non-culturable fungi associated with *A. mexicana* roots, shoots, and rhizospheric soil to understand their possible contribution to acaricide production (Fadiji and Babalola, 2020).

## 2 Materials and methods

### 2.1 Sample sites and collection methods

The roots and shoots of healthy *A. mexicana* and its rhizospheric soil samples were collected during the flowering stage from the bank of the Kopili River, with the locations coded as L1 (26.12° N, 92.27° E) in Amlighat, Morigaon district, and L2 (26.70° N, 93.97° E) in Dergaon, Golaghat district, Assam, India. For each location, sampling was performed at three different sites. The soil samples were collected according to Luster et al. (2008). The samples were coded as follows: L1 root (L1R), L1 shoot (L1S), L1 rhizospheric soil (L1RS), L2 root (L2R), L2 shoot (L2S), and L2 rhizospheric soil (L2RS) samples of *A. mexicana*. All sample collections and experimental procedures were carried out in triplicate to ensure the accuracy and reproducibility of the findings. Detailed information regarding the samples is provided in **Supplementary Table S1**. Fresh samples were stored in liquid nitrogen until used.

### 2.2 Metagenomic DNA isolation and amplicon/library preparation

The metagenomic DNA from rhizospheric soil samples was isolated using the Qiagen DNeasy Power Soil kit (Qiagen, USA). The CTAB method was used for DNA extraction from root and shoot samples (Zhang et al., 2010). The concentration of metagenomic DNA was measured using a Qubit Fluorimeter (ThermoFisher Scientific, India) (version 3.0). The universal primers ITS3F (5’-GCATCGATGAAGAACGCAGC-3’) and ITS4R (5’-TCCTCCGCTTATTGATATGC-3’) were used for amplification of the internal transcribed spacer 2 (ITS2) region from extracted metagenomic DNA. The amplicons (~250 bp) were selected from the agarose gel using a Qiagen gel elution kit (Qiagen, USA). The 5 ng of amplified product from each sample was used for DNA library preparation using the NEB Next Ultra DNA library preparation kit (New England Biolabs, India). DNA library quality and quantity were estimated using the Agilent 2200 Tape Station (Agilent Technologies, US). The prepared DNA libraries were sequenced on an Illumina HiSeq 2500 (Illumina, Inc., America) using 2 × 250-cycle chemistry at Agri Genome Labs Private Limited, Kerala, India.

### 2.3 Sequence quality check and identification of ITS region

The FastQC tool (version 0.11.8) was used with default parameters to check the sequence quality of the samples. The base composition, base quality, guanine-cytosine (GC) content, adapter dimers, and uncertain bases were thoroughly checked initially for the analysis. Raw reads produced by the sequencer might include artificial reads from adapter dimers during the DNA library construction. Consequently, to obtain a high-quality clean-read dataset, the following steps were carried out: (i) removal of reads that may have resulted from adapter dimer contamination; (ii) elimination of reads with an average Phred quality score below Q30, which was considered the minimum score for a high-quality base; (iii) deletion of reads with a significant excess of “N” (≥5% of the read). The nucleotide composition of the sequence reads was checked for all samples. The three steps were carried out for the identification of the ITS region from the paired-end reads: (i) trimming of sequencing primers using an in-house Perl script; (ii) building the consensus ITS region from trimmed paired-end reads using the FLASH program (version 1.2.11); and (iii) alignment of the obtained sequences. Pre-processing analysis of reads was performed to remove chimeras using *de-novo* chimera removal method UCHIME (version 11) in the VSEARCH tool (Caporaso et al., 2010; Luster et al., 2008; Zhang et al., 2010; Rognes et al., 2016).

### 2.4 Taxonomy categorization

Operational taxonomic unit (OTU) selection and taxonomy categorization were performed using preprocessed consensus ITS region sequences. Preprocessed reads from all six samples were collected and grouped into OTUs based on the similarity of their sequences using the Uclust program available in QIIME software. The QIIME 1 program was used for the complete downstream analysis. The obtained clean sequences from each clustered OTU were identified and aligned against the ITS region database using the PyNAST program (Shin et al., 2016). Taxonomy was assigned to the OTUs, and read abundances were identified for all samples. Further, taxonomic classification was performed using the Ribosomal Database Project classifier by mapping each representative sequence against the UNITE fungal reference database.

The relative abundance plots of the samples were made on the basis of OTUs at the phylum, class, order, family, genus, and species levels. Alpha diversity of all samples was investigated using rarefaction curves and the Chao1, Simpson, and Shannon diversity indices. Beta diversity was determined using heatmaps, Venn diagrams, principal component analysis (PCA), and weighted and unweighted UniFrac metrics. Network co-occurrence of the fungal microbiome was analyzed at the phylum and species levels.

### 2.5 Statistical analysis

The analysis of variance (ANOVA) was performed using SPSS 13.0 (IBM, Armonk, NY, USA) to calculate differences in relative abundance, and p < 0.05 was considered statistically significant.

### 2.6 GenBank accession numbers

NGS data were submitted to the BioProject database at NCBI under accession numbers PRJNA728854, PRJNA728856, PRJNA728860, PRJNA728865, and PRJNA728868.

## 3 Results

The interactions of the fungal communities associated with the rhizospheric soil (L1RS, L2RS), roots (L1R, L2R), and shoots (L1S, L2S) of *A. mexicana* from two different geographical regions of Northern India were studied using mNGS technology. The ITS region was amplified and sequenced from the total metagenomic DNA extracted from all samples of *A. mexicana*. Our dataset allows comparison of fungal metagenomic data from rhizospheric soil, roots, and shoots between the two locations (L1 and L2).

### 3.1 Illumina library preparation

The Illumina Seq 2500 was used for sequencing the amplified metagenome. The raw read statistics and sequence quality calculations are presented in **Supplementary Table 1**. The raw FASTQ reads were paired-end reads and were obtained for each sample. Among all samples, the highest numbers of raw FASTQ reads were observed in the L2 samples (L2R (318,873), L2S (233,114), and L2RS (878,701)) compared with the L1 samples, as summarized in **Supplementary Table 1**. Slight variation in GC% was observed among different samples (**Supplementary Table 1**). The GC content varied from 50.86% to 56.65% among the samples. In the Phred score distribution plot, over 80% of the total reads exhibited a Phred score greater than 30, which implies an error probability >=0.001. Phred quality scores of all the samples suggested that the total paired-end reads had more than 99.9% base call accuracy. Significantly lower fungal richness was observed in L1 samples than in L2 samples.

Nevertheless, fungal diversity was shown to be affected by geographic location. In particular, we observed that L1 samples had much lower fungal richness than L2 samples. These data suggest that geographical origin, the rhizosphere, and the phyllosphere all influence microbial richness.

### 3.2 Alpha diversity

Alpha diversity demonstrates the variation in fungal community structure among root, shoot, and rhizospheric soil samples of *A. mexicana*. The total number of operational taxonomic units (OTUs) varied among individual samples (**Supplementary Figures 1A, 2A**). The results revealed that the fungal phylotypes ranged from 272 ± 65.39 (lowest in L1R) to 2,413.33 ± 186.57 OTUs (highest in L2RS) in the samples (**Table 1**). The Shannon metric is a measure of fungal diversity in a particular area. A wide range of unique species was observed in the samples according to the Shannon index (**Supplementary Figures 1B, 2B**; **Table 1**). L2RS (5.84 ± 0.41) showed the highest fungal species richness, followed by L1RS (5.65 ± 0.39). The highest fungal community richness (Chao1 index value) was observed in L2RS (2,848.33 ± 215.37), followed by L1RS (2,715 ± 252) (**Supplementary Figures 1C, 2C**; **Table 1**). As evident from **Table 1**, the number of OTUs, the Chao1 index, phylogenetic diversity, and the Shannon index of fungal species were higher in L2 samples than in L1 samples. Further, the Simpson index value was higher in L1 samples (L1S and L1R) than in L2 samples (L2S, L2R), except for L2RS samples, which showed the highest Simpson index (**Supplementary Figures 1D, 2D**).

**Table 1:** Summary of NGS data processing and the alpha diversity of shoot, root, and rhizospheric soil samples of *A. mexicana* from Amlighat (L1) and Dergaon (L2).

Codes	Preprocessed consensus sequences	Number of valid sequences in each sample	OTU	Chao1	Shannon	Phylogenetic diversity	Simpson
L1S	157,295.33 ± 9,612.88	155,410 ± 9,612.88	306 ± 45.90	381.30 ± 46.87	3.01 ± 0.25	50.97 ± 7.16	0.80 ± 0.02
L2S	199,510.67 ± 38,054.39	166,510 ± 0.00	307.67 ± 79.10	407.79 ± 32.73	2.84 ± 0.45	55.06 ± 11.76	0.78 ± 0.04
L1R	148,523.67 ± 16,243.36	147,640 ± 16,677.73	272 ± 65.39	379.45 ± 86.15	3.15 ± 0.27	49.43 ± 5.84	0.83 ± 0.02
L2R	166,806.67 ± 12,491.72	161,515 ± 8,651.59	362.33 ± 34.49	462.82 ± 24.91	3.17 ± 0.30	61.36 ± 2.44	0.81 ± 0.03
L1RS	413,763 ± 18,246	405,195 ± 19,097	2,310 ± 285	2,715 ± 252	5.65 ± 0.39	306.65 ± 41.81	0.94 ± 0.01
L2RS	564,816 ± 12,036	458,710 ± 0	2,413.33 ± 186.57	2,848.33 ± 215.37	5.84 ± 0.41	397.27 ± 59.25	0.95 ± 0.02

Note* Values are means ± standard deviations for the replicates. L denotes location, L1 = Amlighat, L2 = Dergaon, S = shoot, R = root, and RS = rhizospheric soil samples.

### 3.3 Beta diversity

Beta diversity analyzes similarities and dissimilarities between two or more communities. NGS mainly identified a total of 5 phyla, 32 classes, 39 orders, 68 families, 125 genera, and 273 species. It is evident from the Venn diagram that a total of 5,328 different types of OTUs were obtained from the rhizospheric soil samples, among which 45.1% were common (**Supplementary Figure S3**). Further, 64.5% and 47.5% of the OTUs were common among the shoot and root samples, respectively, from the two locations. Therefore, the cumulative number of OTU types was higher in L2 than in L1 samples.

The fungal taxanomic composition associated with *A. mexicana* was approximately similar among the six samples, but the relative abundances were notably different (**Supplementary Tables 2–7**; **Figure 1**; **Supplementary Figures 4–8**). Ascomycota and Basidiomycota were the major fungal phyla observed in the samples (**Figure 1**; **Supplementary Table 2**). In addition to these fungal phyla, Zygomycota and unknown taxa were also observed in the samples. Variations in the microbiome depending on the phyllosphere, rhizosphere, and geographical location were apparent when relative abundances at the phylum level were considered. Saccharomycetes was the most abundant fungal class in L1S (25.13%), L2S (20.78%), and L2R (18.42%) samples, while Sordariomycetes was the most abundant class in L1R (27.01%), L1RS (30.60%), and L2RS (30.13%). As evident from **Supplementary Table 3**, the relative abundances of fungal classes were significantly different between the two locations. The order Saccharomycetales was the most abundant, followed by Hypocreales and Microascales (**Supplementary Table 4**). As shown in **Supplementary Table 4**, the relative abundance of the order Incertae_sedis was highest in L1R (14.72%), which was seven times higher than in L2R (2.23%).

**Figure 1:**
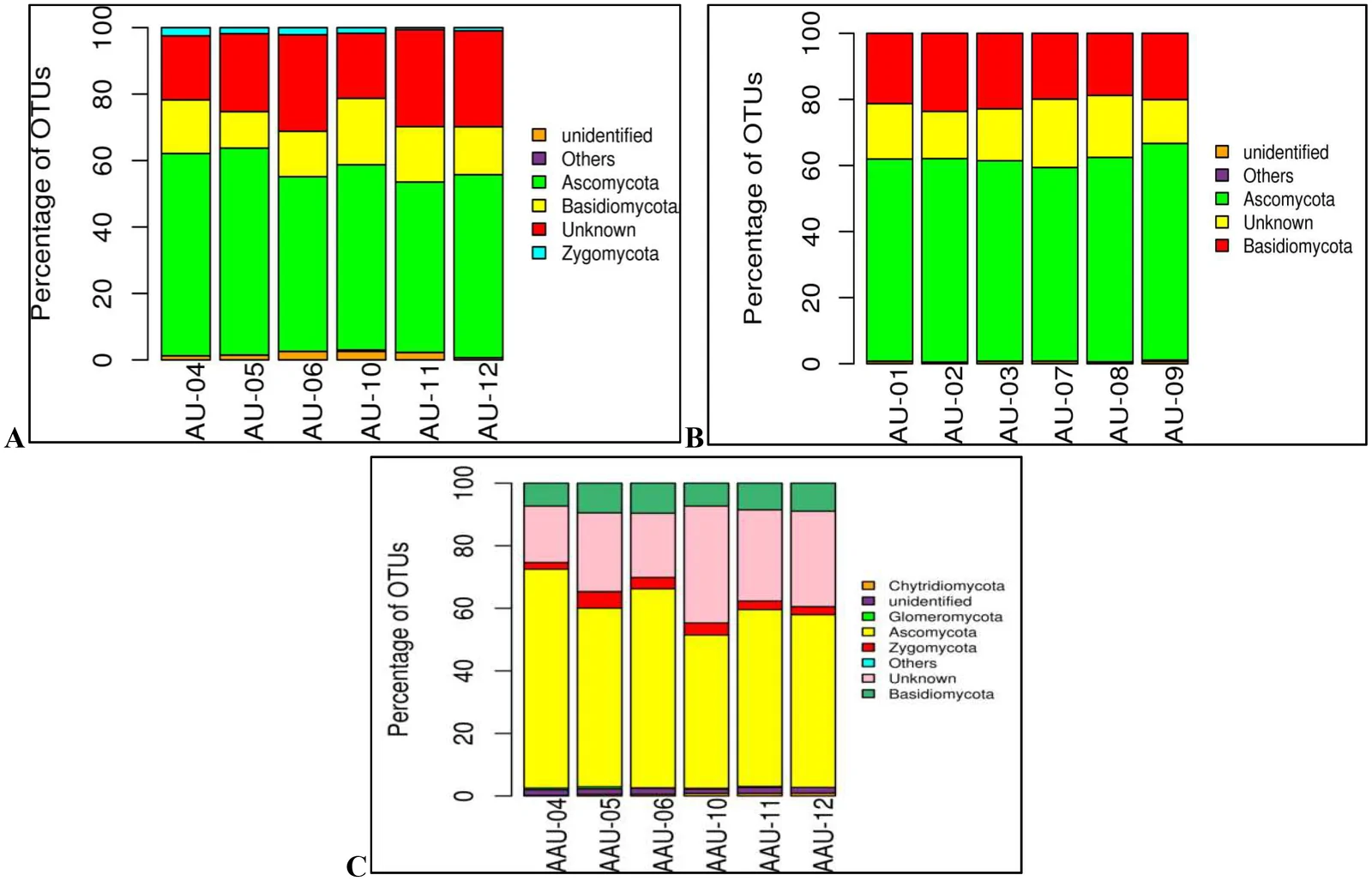
Relative abundance of fungal endophytes at the phylum level (OTUs): **(A)** shoot, **(B)** root, **(C)** rhizospheric soil.

As demonstrated in **Supplementary Table S5**, *Debaryomycetaceae* was the dominant family in L1S, with a relative abundance of 25.06%, which was 1.20 times higher than that in L2S (20.72%). *Debaryomycetaceae* was followed by *Ceratocystidaceae*, *Ganodermataceae*, *Mycosphaerellaceae*, and *Filobasidiaceae*, with relative abundances of 5.15%, 2.73%, 2.50%, and 1.04%, respectively, in L1S. In L2S, *Ceratocystidaceae* (7.61%), *Ganodermataceae* (2.25%), *Mycosphaerellaceae* (1.44%), and *Hypocreaceae* (1.27%) were observed.

Moreover, the relative abundance of the Nectriaceae family in L1R was 11.73%, which was 3.23 times higher than that in L2R (3.63%) (**Supplementary Table 5**). The second most abundant family was Incertae_sedis, with a relative abundance of 7.93%, which was 10.16 times higher than that in L2R (0.78%), followed by *Plectosphaerellaceae* (6.71%), *Phyllachoraceae* (5.85%), and *Ceratocystidaceae* (1.84%). However, in L2R, the dominant group was *Debaryomycetaceae* (18.36%), which was four times higher than that in L1R, followed by *Phyllachoraceae* (5.87%), *Ceratocystidaceae* (2.56%), *Mycosphaerellaceae* (2.38%), and *Ganodermataceae* (1.46%). *Debaryomycetaceae* was also dominant in L1RS and L2RS, with relative abundances of 21.69% and 22.99%, respectively. *Nectriaceae* (20.07%) and *Trichocomaceae* (14.61%) were the second and third most abundant families, respectively, in the L1RS samples. Similarly, *Nectriaceae* was the second most dominant family in L2RS, with a relative abundance of 14.10%.

*Meyerozyma* was the leading genus in all samples, with relative abundances of L1S (25.06%), L1R (10.46%), L1RS (21.69%), L2S (20.72%), L2R (18.36%), and L2RS (17.70%). In addition, the genus *Ceratocystis* occurred in all samples at different relative abundances. As shown in **Supplementary Table S6**, the L1RS sample is the richest sample in terms of fungal genera, and L2RS is the second richest sample. A similar result was observed at the species level. *Ceratocystis paradoxa* was a common fungus in all samples, with varying relative abundances (**Supplementary Table 7**). It was the most abundant species in L1S, L2S, and L2RS samples.

Krona plots were used for analysis of the taxonomic hierarchy of the fungi. The Krona plots were made on the basis of phylum, class, order, family, genus, and species for all six samples (**Figure 2**). Only reads from dominant fungi were selected for generating these plots. In all the samples, the most abundant phylum was Ascomycota followed by Basidiomycota. Fifty-four percent of all the fungal reads were unclassified for root and shoot samples. Whereas, only 16% of all the fungi was unclassified in rhizospheric soil samples, which is 3.37% less then root and shoot samples.

**Figure 2:**
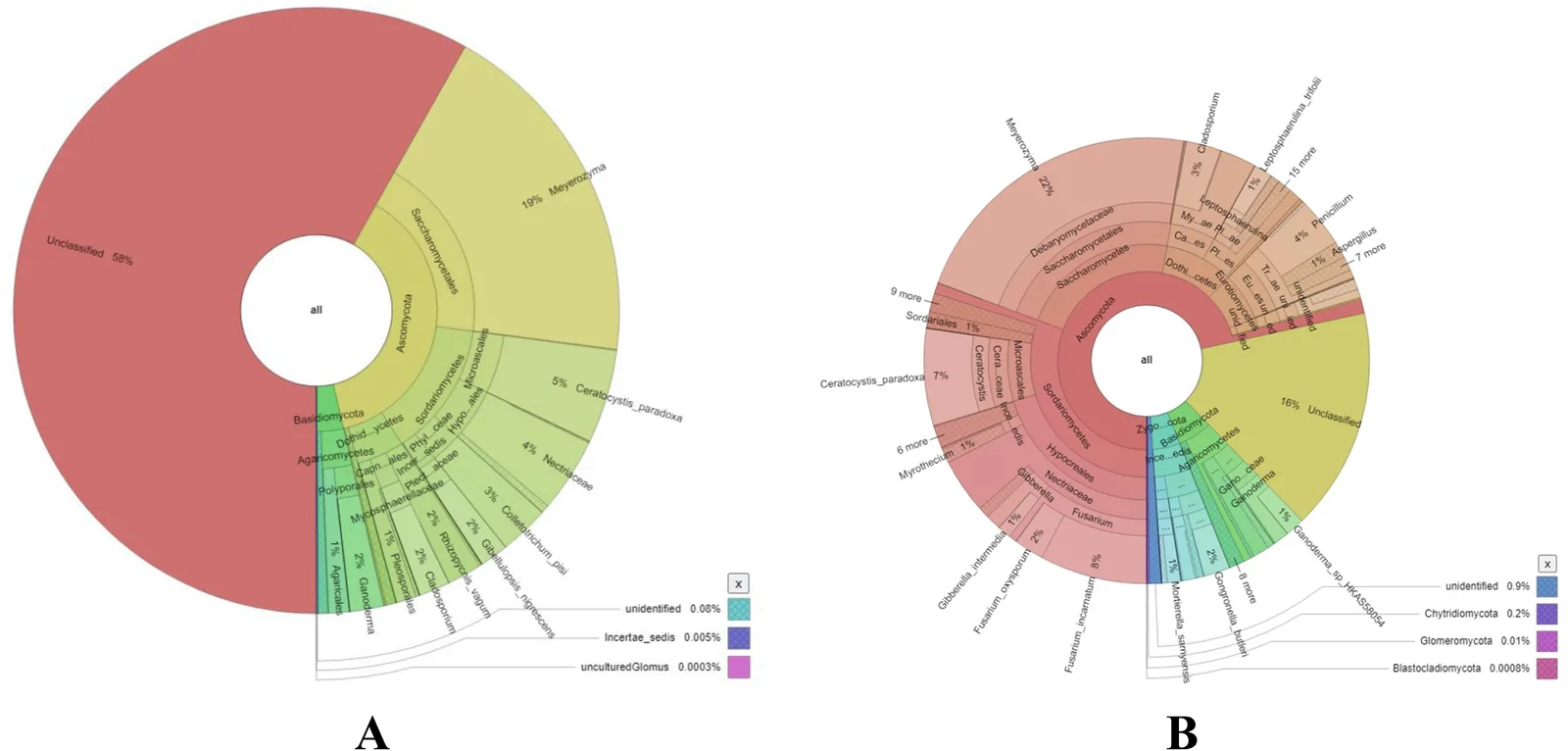
Krona plot representing endophytic fungal taxa of *A. mexicana*. **(A)** Root and shoot; **(B)** rhizospheric soil.

In this study, we observed that the PCA plots showed the greatest variation at the phylum level in root and shoot samples, with contributions from PC1 (42.9%) and PC2 (28.6%) (**Supplementary Figure 9**). According to **Figure 2B**, fungal communities were slightly similar in the rhizospheric soils of L1 and L2, with contributions from PC1 (72.3%) and PC2 (18.8%) (**Supplementary Figure 9b**).

Distance matrix calculation was determined by hierarchical clustering analysis. The hierarchical cluster trees were generated using the OTUs observed in the samples and the weighted and unweighted UniFrac algorithms. The sequences can form clusters according to their similarities to other sequences in a source database, and OTUs can be generated by clustering sequences *de novo* (Liu et al., 2008; Sun et al., 2012). A jackknife test was used to generate a consensus unweighted pair group method with arithmetic mean (UPGMA) tree using the unweighted UniFrac distance matrix for all samples. The UPGMA clustering based on Bray–Curtis distance showed a similar fungal community structure amongst the three replicates in each sample and apparent variations among all samples (**Supplementary Figure 10**). According to unweighted UniFrac analysis, shoots (L1S and L2S), roots (L1R and L2R), and rhizospheric soil (L1RS and L2RS) had similar species; dissimilarities were also noted in the respective samples. Based on the heatmap, *Ganoderma* sp., *Meyerozyma* sp., and *Ceratocystis* sp. were abundant in all samples. *Cladosporium* sp., *Fusarium* sp., *and Penicillium* sp. were present in L1RS and L2RS samples. *Rhodotorula* sp., *Trichoderma* sp., *Lasiodiplodia* sp., *Hypocreales* sp., *Rhizopycnis* sp., *and Sporobolomyces* sp. were detected in shoot and root samples at high abundances; however, L1RS and L2RS did not show these fungal species. Unknown fungi were identified in high abundances in all six samples from the two locations (**Figures 3**, **4**). We observed variation at the species level in all samples, which may contribute to differences in the production of acaricidal compounds in *A. mexicana* plants between L1 and L2. In a collaborative study, higher levels of the acaricidal compounds Berberine and sanguinarine were observed in L1 samples than in L2 samples (Upadhaya et al., 2020).

**Figure 3:**
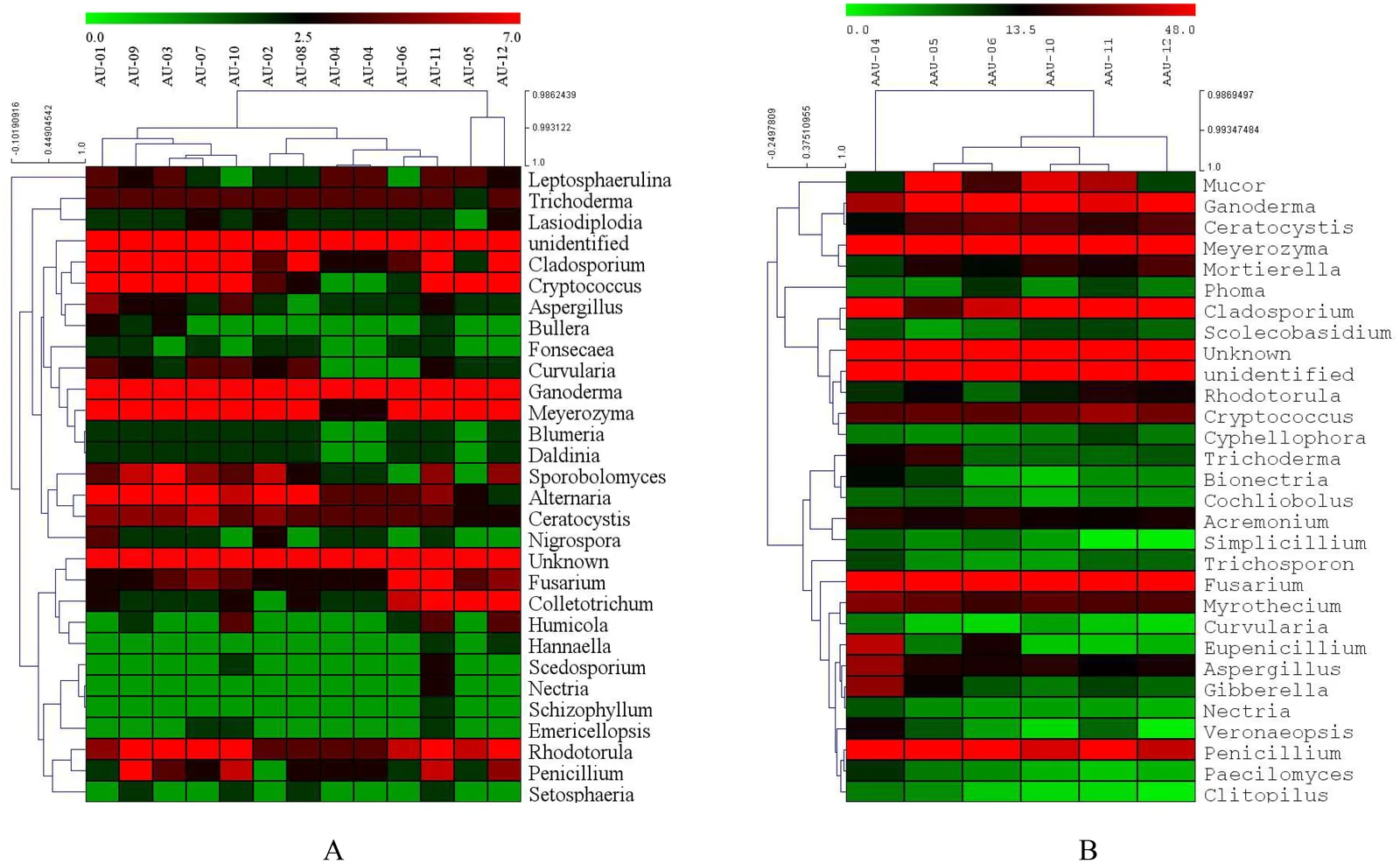
Heatmap of the abundance of the fungal taxa identified at the genus level by NGS analysis of each sample, including the replicates, from *A. mexicana*. Higher intensities of the color indicate higher abundances of the isolates. **(A)** Root and shoot; **(B)** rhizospheric soil.

**Figure 4:**
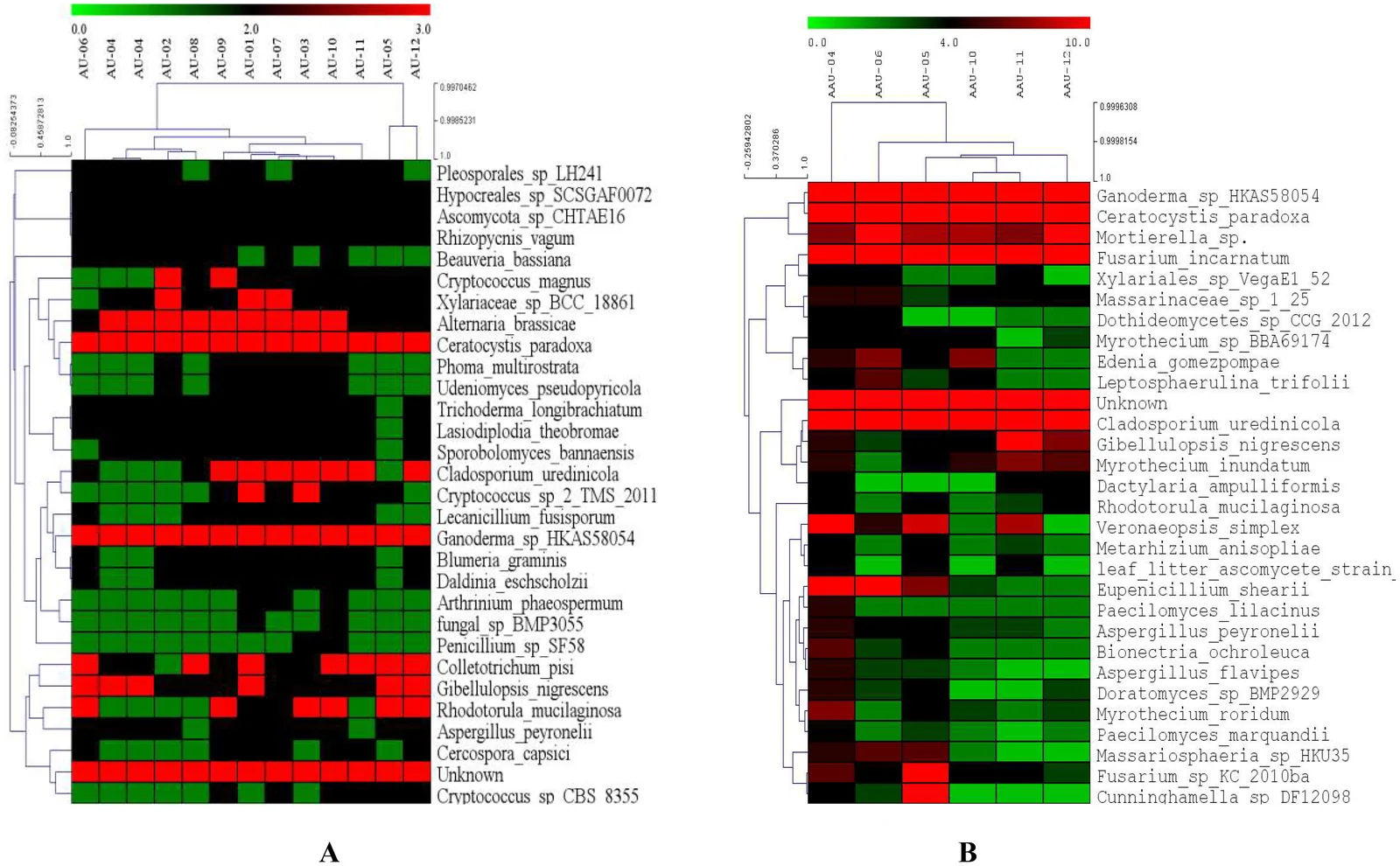
Heatmap of fungal species abundance identified by NGS analysis of each sample, including the replicates. Color intensities are directly proportional to the abundance of the isolates. **(A)** Root and shoot, **(B)** rhizospheric soil.

The co-occurrence network analysis exhibited relationships among the fungal communities in L1 and L2 samples. The phylum Ascomycota was the most abundant in all six samples, followed by Basidiomycota (**Supplementary Figure 11**). The phylum Zygomycota was observed only in rhizospheric samples. The co-occurrence network analysis showed that the complexity of fungal diversity was highest in rhizospheric samples, as indicated by the highest number of nodes and edges (**Supplementary Figures 11–13**).

## 4 Discussion

To the best of our knowledge, the composition and diversity of fungal communities were evaluated for the first time in the roots and shoots of *A. mexicana* from the northeastern region (NER) of India using the high-throughput mNGS technique. The number of fungal species discovered has increased rapidly with the development of mNGS methods. According to Blackwell (2011), approximately 5.1 million fungi are known to date. However, Kaul et al. (2016) pointed out that only approximately 10,000 fungal species have been classified and analyzed; the identification and characterization of the majority of fungi remain unknown. The NER of India is an “untouched place” and is also considered a “biodiversity hotspot” due to the diversity of fauna and flora. The highlands of the Northeast and their climate are best suited for the growth of different species of plants and animals. The novelty of the study lies in determining similarities and dissimilarities in fungal populations in samples collected from two different locations in Assam, L1 and L2. In our earlier collaborative study, Upadhaya et al. (2020) tested samples collected from the northeastern region of India against ticks. They found variations in acaricidal properties in samples collected from different locations in the NER. They found the highest acaricidal properties {80%–100% (95.0% ± 2.7 mean ± SE) mortality range} in Amlighat and the lowest {10%–40% (25.0% ± 8.6 mean ± SE) mortality range} in Dergaon. Therefore, these two locations were selected for the present study. Differences in rhizospheric and endophytic fungal populations may contribute towards the observed change in acaricidal activity of the samples. Therefore, we examined the variation in the fungal populations in plant tissues (root and shoot) and rhizospheric soil. As expected, we found different fungal diversity in the samples, which may contribute to the variation in acaricidal properties. We also isolated endophytes of *A. mexicana* from two locations in the NER of India, Amlighat and Dergaon, and further tested them for their ability to protect *Arabidopsis thaliana* against the pathogen *Pseudomonas syringae pv tomato* DC3000 (Singh et al., 2021).

The number of OTUs, Chao1 indices, and phylogenetic diversity were lower in L1 samples than in L2 samples, implying lower fungal richness in L1 samples. However, the Shannon and Simpson indices were higher in L1 samples than in L2 samples. The variation in plant populations may change soil microhabitats, resulting in alterations in soil fungal community structure (Delgado-Baquerizo et al., 2018). According to previous studies, fungal community structure and function depend on rhizospheric soil (Shen et al., 2020). *Meyerozyma* is the most abundant and commonly found genus in all four samples. It was found greater in L1 samples (L1S 25.06% and L1RS 21.69%) and is known to have antimicrobial ability against human pathogenic bacteria.

In the present study, the phylum Ascomycota was predominantly observed in all samples. Our results demonstrated that Ascomycota (37.47%–75.84%) and Basidiomycota (3.53%–6.95%) were the dominant fungal groups in the plant samples from L1 and L2. However, Ascomycota was more abundant in L1 (37.41%–75.84%) than in L2 (38.06%–67.98%); a similar result was observed for Basidiomycota. These findings correlate with studies indicating that the soil environment contains a wide and complex microbial composition, which makes it easier for other fungi to colonize the roots and then move to the phyllosphere (Afzal et al., 2019; Trujillo et al., 2014; Zhang et al., 2018).

The members of the phylum Ascomycota are key producers of pharmaceutical and commercially important secondary metabolites. Studies have shown that the fungus *Metarhizium anisopliae* of the phylum Ascomycota is used for its acaricidal properties against *Ixodes scapularis* (deer tick), *Rhipicephalus microplus* (cattle tick), *Argas persicus* (fowl tick), and *Argas reflexus* (pigeon tick) (Benjamin et al., 2002; Walter et al., 2020; Pourseyed et al., 2010; Tavassoli et al., 2011). In addition to this property, members of Ascomycota are cellulose and lignin decomposers and are known to produce lipases, α-amylases, probiotics, statins, and compounds with antioxidant, antidiabetic, antimicrobial, anticancer, antiviral, and antiprotozoal activities (Janusz et al., 2017; Frac et al., 2018; Snelders et al., 2021; Tian et al., 2015; Ma et al., 2017; Ibrahim et al., 2015; Dewi et al., 2015; de Carvalho et al., 2019).

In our previous study, *Aspergillus* and *Penicillium* species belonging to Ascomycota were isolated from *the A. mexicana* plant (Singh et al., 2023, 2020). The phylum Ascomycota contains some pharmaceutically important fungal species that can produce medicinal compounds. Teimoori-Boghsani et al. (2020) reported that caffeic acid was detected in the secondary metabolite profile of fungal isolates belonging to the genera *Paraphoma* and *Talaromyces*. The phenolic compound (caffeic acid) has been reported in endophytic fungi *Cladosporium velox*, *Penicillium canescens*, and *Fusarium chlamydosporum* (Singh et al., 2016). Caffeic acid is an important phenolic compound of *A. mexicana* and is known for its antibacterial, antifungal, and modulatory effects (Lima et al., 2016). The fungal isolates *Cladosporium*, *Penicillium, Talaromyces*, and *Fusarium* were observed in all samples. Verma et al. (2014) reported that *Trichoderma* species can alter the production of the acaricidal compound sanguinarine in *Papaver somniferum* (Verma et al., 2014). In the present study, *Trichoderma* species were present in all samples at different abundances. *P. somniferum* and *A. mexicana* belong to the same plant family, *Papaveraceae*, and both produce sanguinarine. Therefore, it may be possible that endophytic and arbuscular mycorrhizal fungi are responsible for the production and alteration of acaricidal compounds in our study system (*A. mexicana*).

Our results showed that the relative abundance of the second most abundant phylum, Basidiomycota, in L2 samples (root 3.53%, shoot 3.85%, and rhizospheric soil 6.95%) was significantly higher than in L1 samples. Basidiomycota has similar economic importance in medical and environmental safety. The genus *Septobasidium*, which belongs to Basidiomycetes, is able to produce acaricidal compounds against scale ticks (Rao and Narladkar, 2018).

## 5 Conclusion

In conclusion, the results of the current study revealed that fungal communities associated with plant tissues (root and shoot) and rhizospheric soil were identified at two different locations, Amlighat (L1) and Dergaon (L2), in Assam, India, using next-generation sequencing. A higher number of OTUs was found in L2 samples. The predominant phylum in all samples was Ascomycota (37.47%–75.84%), followed by Basidiomycota (3.53%–6.95%). Every sample contained *Trichoderma* species, albeit at varying relative abundances. *A. mexicana* is a well-known medicinal plant containing pharmaceutically important secondary metabolites such as sanguinarine, berberine, jatrorrhizine, protopine, and columbamine. Reports show that *Trichoderma* species can alter sanguinarine production in *P. somniferum*, which belongs to the same family as *A. mexicana*, Papaveraceae. Similarly, *Trichoderma* species and other fungi present in the samples may contribute to the production of secondary metabolites in *A. mexicana*.

## Data Availability

The original contributions presented in the study are publicly available. This data can be found here: NCBI BioProject PRJEB127367, Indian Nucleotide Data Archive (INDA) accession INRP000755.

## References

[B1] Afzal I. Shinwari Z. K. Sikandar S. Shahzad S. (2019). Plant beneficial endophytic bacteria: mechanisms, diversity, host range and genetic determinants. Microbiol. Res. 221, 36–49. doi: 10.1016/j.micres.2019.02.001 30825940

[B2] Alawiye T. T. Babalola O. O. (2019). Bacterial diversity and community structure in typical plant rhizospheric. Divers 11, 179. doi: 10.3390/d1110017930654563

[B3] Alharbi S. M. Al-Sulami N. Al-Amrah H. Anwar Y. Gadah O. A. Bahamdain L. A. . (2025). Metagenomic characterization of the Maerua crassifolia soil rhizosphere: Uncovering microbial networks for nutrient acquisition and plantresilience in arid ecosystems. Genes (Basel). 16, 285. doi: 10.3390/genes16030285 40149437 PMC11942469

[B4] Benjamin M. A. Zhioua E. Ostfeld R. S. (2002). Laboratory and field evaluation of the entomopathogenic fungus *Metarhizium anisopliae* (Deuteromycetes) for controlling questing adult *Ixodes scapularis* (Acari: Ixodidae). J. Med. Entomol. 39, 723–728. doi: 10.1603/0022-2585-39.5.723 12349854

[B5] Bhadra F. Vasundhara M. (2024). Anti-inflammatory potential of aconitine produced by endophytic fungus *Acremonium alternatum*. World J. Microbiol. Biotechnol. 40, 274. doi: 10.1007/s11274-024-04083-y 39030384

[B6] Bhadra F. Vasundhara M. (2025). Lipoxygenase inhibitor from endophytic fungus Cladosporium ramotenellum associated with *Argemone mexicana* L. Chem. Biodivers. 22, e01400. doi: 10.1002/cbdv.202501400 40921043

[B7] Blackwell M. (2011). The fungi: 1, 2, 3 . 5.1 million species? Am. J. Bot. 98, 426–438. doi: 10.3732/ajb.1000298 21613136

[B8] Brader G. Compant S. Vescio K. Mitter B. Friederike T. F. Li-Jun M. . (2017). Ecology and genomic insights into plant-pathogenic and plant-nonpathogenic endophytes. Annu. Rev. Phytopathol. 55, 61–83. doi: 10.1146/annurev-phyto-080516-035641 28489497

[B9] Bulgarelli D. Schlaeppi K. Spaepen S. (2013). Structure and functions of the bacterial microbiota of plants. Annu. Rev. Plant Biol. 64, 807–838. doi: 10.1146/annurev-arplant-050312-120106 23373698

[B10] Caporaso J. G. Kuczynski J. Stombaugh J. Bittinger K. Bushman F. D. Costello E. K. . (2010). QIIME allows analysis of high-throughput community sequencing data. Nat. Methods 7, 335–336. doi: 10.1038/nmeth.f.303 20383131 PMC3156573

[B11] de Carvalho C. R. Ferreira M. C. Amorim S. S. de Silva Florindo R. H. de Assis J. C. S. Zani C. L. . (2019). “ Bioactive compounds of endophytic fungi associated with medicinal plants,” in Recent Advancement in White Biotechnology Through Fungi. Fungal Biology. Eds. Yadav A. Singh S. Mishra S. Gupta A. ( Springer, Cham). doi: 10.1007/978-3-030-14846-1_11

[B12] De la Cruz-Velueta M. F. Muñoz-Sánchez J. A. Vázquez-Flota F. A. (2024). Isolation of protoplasts from tissues of Mexican prickly poppy (*Argemone mexicana* L.): An alkaloid-producing medicinal plant. Methods Mol. Biol. 2827, 435–443. doi: 10.1007/978-1-0716-3954-2_29 38985287

[B13] Delgado-Baquerizo M. Fry E. L. Eldridge D. J. de Vries F. T. Manning P. Homonts K. . (2018). Plant attributes explain the distribution of soil microbial communities in two contrasting regions of the globe. New Phytol. 219, 574–587. doi: 10.1111/nph.15161 29672854

[B14] Dewi R. T. Tachibana S. Fajriah S. Hanafi M. (2015). α-Glucosidase inhibitor compounds from Aspergillus terreus RCC1 and their antioxidant activity. Med. Chem. Res. 24, 737–743. doi: 10.1016/j.proche.2015.12.02442574925

[B15] Fadiji A. E. Babalola O. O. (2020). Metagenomics methods for the study of plant-associated microbial communities: A review. J. Microbiol. Methods 170, 105860. doi: 10.1016/j.mimet.2020.105860 32027927

[B16] Frac M. Hannula S. E. Bełka M. Jedryczka M. (2018). Fungal biodiversity and their role in soil health. Front. Microbiol. 9, 707. doi: 10.3389/fmicb.2018.00707 29755421 PMC5932366

[B17] Ibrahim S. R. M. Elkhayat E. S. Mohamed G. A. Khedr A. I. M. Fouad M. A. Kotb M. H. R. . (2015). Aspernolides F and G, new butyrolactones from the endophytic fungus Aspergillus terreus. Phytochem. Lett. 14, 84–90. doi: 10.1016/j.phytol.2015.09.00642574925

[B18] Jaiswal J. Siddiqi N. J. Fatima S. Abudawood M. AlDaihan S. K. Alharbi M. G. . (2023). Analysis of biochemical and antimicrobial properties of bioactive molecules of *Argemone mexicana*. Molecules 28, 4428. doi: 10.3390/molecules28114428 37298904 PMC10254925

[B19] Janusz G. Pawlik A. Sulej J. Swiderska-Burek U. Jarosz- Wilkolazka A. Paszczynski A. (2017). Lignin degradation: microorganisms, enzymes involved, genomes analysis and evolution. FEMS Microbiol. Rev. 41, 941–962. doi: 10.1093/femsre/fux049 29088355 PMC5812493

[B20] Kaul S. Sharma T. Dhar M. K. (2016). Omics” tools for better understanding the plant-endophyte interactions. Front. Plant Sci. 7, 955. doi: 10.3389/fpls.2016.00955 PMC4925718 27446181

[B21] Konika T. A. Uddin M. E. Jahan R. Hasan M. N. Shamsuzzaman M. Shamsudeen S. M. (2026). LC/MS-based phytochemical profiling and immunomodulatory evaluation of three medicinal plants with anticancer and antibacterial activities. Chem. Biodivers. 23, e03142. doi: 10.1002/cbdv.202503142 41918395

[B22] Kulshrestha S. Saraswat I. Goel A. (2026). Retrospection of *Argemone mexicana* and its derived analogs as a futuristic approach exhibiting anticancer potential. Mini-Rev. Med. Chem. 16, 1227–1239. doi: 10.2174/0113895575417548251208235008 41863239

[B23] Lima V. N. Oliveira-Tintino C. D. Santos E. S. Morais L. P. Tintino S. R. Freitas T. S. . (2016). Antimicrobial and enhancement of the antibiotic activity by phenolic compounds: gallic acid, caffeic acid and pyrogallol. Microb. Pathog. 99, 56–64. doi: 10.1016/j.micpath.2016.08.004 27497894

[B24] Liu Z. DeSantis T. Z. Andersen G. L. Knight R. (2008). Accurate taxonomy assignments from 16S rRNA sequences produced by highly parallel pyrosequencers. Nucleic Acids Res. 36, e120. doi: 10.1093/nar/gkn491 18723574 PMC2566877

[B25] Liu Y. Shang Y. Wang X. Li X. Yu Z. Zeng Z. . (2025). Metagenomics and *in vitro* growth-promoting experiments revealed the potential roles of mycorrhizal fungus Humicolopsis cephalosporioides and helper bacteria in Cheilotheca humilis growth. Microorganisms 13, 2387. doi: 10.3390/microorganisms13102387 41156846 PMC12566112

[B26] Lou G. Wang J. Hu J. Gan Q. Peng C. Xiong H. . (2021). Sanguinarine: A double-edged sword of anticancer and carcinogenesis and its future application prospect. Anti-Cancer Agents Med. Chem. 21, 2100–2110. doi: 10.2174/1871520621666210126091512 33573577

[B27] Luster J. Göttlein A. Nowack B. Sarret G. (2008). Sampling, defining, characterising and modeling the rhizosphere-the soil science tool box. Plant Soil 321, 457–482. doi: 10.1007/s11104-008-9781-330311153

[B28] Ma X. Nong X. H. Ren Z. Wang J. Liang X. Wang L. . (2017). Antiviral peptides from marine gorgonian-derived fungus Aspergillus sp. SCSIO 41501. Tetrahedron Lett. 58, 1151–1155. doi: 10.1016/j.tetlet.2017.02.00542574925

[B29] Omsatyam Kumar D. Singh L. P. Chatterjee S. (2026). Phytochemical and phytopharmacological insights into *Argemone mexicana*: Integrative approaches in Eastern Indian region as folk medicine. Curr. Drug Res. Rev. 18, 121–136. doi: 10.2174/0125899775362872250319082142 40192041

[B30] Patocka J. Ovando-Martinez M. Navratilova Z. Oleksak P. Ogwu M. C. Nepovimova E. . (2024). *Argemone mexicana* L. (Papaveraceae): chemistry, pharmacology, and toxicology. Phytochem. Rev. 23, 1167–1182. doi: 10.1007/s11101-024-09918-y30311153

[B31] Pourseyed S. H. Tavassoli M. Bernousi I. Mardani K. (2010). *Metarhizium anisopliae* (Ascomycota: Hypocreales): An effective alternative to chemical acaricides against different developmental stages of fowl tick *Argas persicus* (Acari: Argasidae). Vet. Parasitol. 172, 305–310. doi: 10.1016/j.vetpar.2010.05.014 20541868

[B32] Rao G. U. B. Narladkar B. W. (2018). Role of entomopathogenic fungi in tick control: A review. J. Entomol. Zool. Stud. 6, 1265–1269. Available online at: https://www.entomoljournal.com/archives/?year=2018&vol=6&issue=1&ArticleId=3097.

[B33] Rognes T. Flouri T. Nichols B. Quince C. Mahé F. (2016). VSEARCH: A versatile open source tool for metagenomics. PeerJ 4, e2584. doi: 10.7717/peerj.2584 27781170 PMC5075697

[B34] Saraswat I. Goel A. Gupta J. (2025). An in-depth review on *Argemone mexicana* in the management of liver health and liver cancer. Anti-Cancer Agents Med. Chem. 25, 24–34. doi: 10.2174/0118715206307964240821051756 39225208

[B35] Shen Q. Yang J. Su D. Li Z. Xiao W. Wang Y. . (2020). Comparative analysis of fungal diversity in rhizospheric soil from wild and reintroduced *Magnolia sinica* estimated via high-throughput sequencing. Plants 9, 600. doi: 10.3390/plants9050600 32397167 PMC7284792

[B36] Shin J. Lee S. Go M. J. Lee S. Y. Kim S. C. Lee C. H. . (2016). Analysis of the mouse gut microbiome using full-length 16S rRNA amplicon sequencing. Sci. Rep. 6, 29681. doi: 10.1038/srep29681 27411898 PMC4944186

[B37] Shurigin V. Lu X. Khan A. R. Muhammad M. Ullah I. Egamberdieva D. . (2026). Unveiling the plant growth-promoting and antifungal potential of *Melissa officinalis* endophytes: The integrative culture-dependent and metagenomic approaches. Plant Sci. 369, 113182. doi: 10.1016/j.plantsci.2026.113182 42070688

[B38] Singh V. K. Kumar A. (2023). Secondary metabolites from endophytic fungi: Production, methods of analysis, and diverse pharmaceutical potential. Symbiosis 8, 1–15. doi: 10.1007/s13199-023-00925-9 37360552 PMC10249938

[B39] Singh P. Sharma A. Bordoloi M. J. Nandi S. P. (2020). Molecular identification of endophytic fungi isolated from medicinal plant. Biointerface Res. Appl. Chem. 10, 6436–6443. doi: 10.33263/BRIAC105.64366443

[B40] Singh P. Sharma A. Bordoloi M. J. Nandi S. P. (2023). Antimicrobial, antioxidant, GC-MS analysis and molecular docking analysis of bioactive compounds of endophyte *Aspergillus flavus* from *Argemone mexicana*. J. Microbiol. Biotechnol. Food Sci. 13, e9970. doi: 10.55251/jmbfs.9970

[B41] Singh B. Sharma P. Kumar A. Chadha P. Kaur R. Kaur A. (2016). Antioxidant and *in vivo* genoprotective effects of phenolic compounds identified from an endophytic *Cladosporium velox* and their relationship with its host plant *Tinospora cordifolia*. J. Ethnopharmacol. 194, 450–456. doi: 10.1016/j.jep.2016.10.018 27721051

[B42] Singh P. Sharma A. Nandi S. P. Nandi A. K. (2021). Endophytes from *Argemone mexicana* and *Datura metel* activate induced-systemic resistance in multiple hosts and show specificity in protection. J. Plant Biochem. Biotechnol. 30, 1016–1019. doi: 10.1007/s13562-021-00734-530311153

[B43] Snelders N. C. Petti G. C. van den Berg G. C. M. Seidl M. F. Thomma B. P. H. J. (2021). An ancient antimicrobial protein co-opted by a fungal plant pathogen for in planta mycobiome manipulation. Proc. Natl. Acad. Sci. U.S.A. 118, e2110968118. doi: 10.1073/pnas.2110968118 34853168 PMC8670511

[B44] Strobel G. A. (2003). Endophytes as sources of bioactive products. Microbes Infect. 5, 535–544. doi: 10.1016/S1286-4579(03)00073-X 12758283

[B45] Strobel G. Daisy B. (2003). Bioprospecting for microbial endophytes and their natural products. Microbiol. Mol. Rev. 67, 491–502. doi: 10.1128/MMBR.67.4.491-502.2003 14665674 PMC309047

[B46] Sun Y. Cai Y. Huse S. M. Knight R. Farmerie W. G. Wang X. . (2012). A large-scale benchmark study of existing algorithms for taxonomy-independent microbial community analysis. Brief Bioinform. 13, 107–121. doi: 10.1093/bib/bbr009 21525143 PMC3251834

[B47] Tavassoli M. Pourseyed S. H. Ownagh A. Bernousi I. Mardani K. (2011). Biocontrol of pigeon tick *Argas reflexus* (Acari: Argasidae) by entomopathogenic fungus *Metarhizium anisopliae* (Ascomycota: Hypocreales). Braz. J. Microbiol. 42, 1445–1452. doi: 10.1590/S1517-838220110004000030 24031777 PMC3768737

[B48] Teimoori-Boghsani Y. Ganjeali A. Cernava T. Muller H. Asili J. Berg G. (2020). Endophytic fungi of native *Salvia abrotanoides* plants reveal high taxonomic diversity and unique profiles of secondary metabolites. Front. Microbiol. 10, 3013. doi: 10.3389/fmicb.2019.03013 32010087 PMC6978743

[B49] Tian Y. Q. Lin X. P. Wang Z. Zhou X. F. Qin X. C. Kaliyaperumal K. . (2015). Asteltoxins with antiviral activities from the marine sponge-derived fungus Aspergillus sp. SCSIO XWS02F40. Molecules 21, E34. doi: 10.3390/molecules21010034 26712735 PMC6272915

[B50] Toju H. Tanabe A. S. Sato H. (2018). Network hubs in root-associated fungal metacommunities. Microbiome 6, 116. doi: 10.1186/s40168-018-0497-1 29935536 PMC6015470

[B51] Trujillo M. E. Bacigalupe R. Pujic P. Igarashi Y. Benito P. Riesco R. . (2014). Genome features of the endophytic actinobacterium Micromonospora lupini strain Lupac 08: on the process of adaptation to an endophytic life style? PloS One 9, e108522. doi: 10.1371/journal.pone.0108522 25268993 PMC4182475

[B52] Upadhaya D. Kumar B. Kumar S. Sharma A. K. Fular A. Bisht N. . (2020). Characterization of acaricide resistance in *Rhipicephalus microplus* populations infesting cattle in northeastern India and assessment of local plant extracts for tick management. Vet. Parasitol. 277, 109011. doi: 10.1016/j.vetpar.2019.109011 31841946

[B53] Verma P. Khan S. A. Mathur A. K. Ghosh S. Shanker K. Kalra A. (2014). Improved sanguinarine production via biotic and abiotic elicitations and precursor feeding in cell suspensions of latex-less variety of Papaver somniferum with their gene expression studies and upscaling in bioreactor. Protoplasma 251, 1359–1371. doi: 10.1007/s00709-014-0638-8 24677097

[B54] Walter O. B. Rafael L. R. Markus B. Caio J. B. C. R. Marilene H. V. Augusto S. . (2020). Updating the application of *Metarhizium anisopliae* to control cattle tick *Rhipicephalus microplus* (Acari: Ixodidae). Exp. Parasitol. 208, 107812. doi: 10.1016/j.exppara.2019.107812 31809704

[B55] Yang S. Johnson M. A. Hansen M. A. Bush E. Li S. Vinatzer B. A . (2022). Metagenomic sequencing for detection and identification of the boxwood blight pathogen *Calonectria pseudonaviculata*. Sci. Rep. 12, 1399. doi: 10.1038/s41598-022-05381-x 35082361 PMC8791934

[B56] Zhang B. M. Wang Z. B. Xin P. Wang Q. H. Bu H. Kuang H. X. (2018). Phytochemistry and pharmacology of genus Ephedra. Chin. J. Nat. Med. 16, 811–828. doi: 10.1016/S1875-5364(18)30123-7 30502763

[B57] Zhang Y. J. Zhang S. Liu X. Z. Wen H. A. Wang M. (2010). A simple method of genomic DNA extraction suitable for analysis of bulk fungal strains. Lett. Appl. Microbiol. 51, 114–118. doi: 10.1111/j.1472-765X.2010.02867.x 20536704

[B58] Zhong F. Chen Y. Chen J. Liao H. Li Y. Ma Y. (2022). Jatrorrhizine: A review of sources, pharmacology, pharmacokinetics and toxicity. Front. Pharmacol. 12, 783127. doi: 10.3389/fphar.2021.783127 35095493 PMC8793695

