# Supplementary material for: Dominance of ascomycota and ubiquity of Trichoderma in Argemone mexicana microbiomes across Assam, India
Source: Front Fungal Biol. 2026 Oct 8;7:1868321. doi: 10.3389/ffunb.2026.1868321 (PMC13687004; doi:10.3389/ffunb.2026.1868321)
Supplement: Supplementary file 1 [file SupplementaryFile1.docx]

**Supplementary files**

**Supplementary Tables:**

**Table S1** Raw reads statistics and sequence quality measurement of metagenomic data of microbes associated with *A. mexicana*.

| Codes | Location | Tissue | Read size (bp) | Total Paired-end reads | GC% | Phred score |
| --- | --- | --- | --- | --- | --- | --- |
| AU1 | L1 | Shoot | 250 | 247484 | 54.28 | 37.13 |
| AU2 | L1 | Shoot | 250 | 224509 | 54.45 | 37.11 |
| AU3 | L1 | Shoot | 250 | 226408 | 54.39 | 37.38 |
| L1S | Mean ± SD |  |  | **232800 ±12751** | **54.37** | **37.20** |
| AU4 | L1 | Root | 250 | 190474 | 55.10 | 37.29 |
| AU5 | L1 | Root | 250 | 201454 | 58.09 | 37.28 |
| AU6 | L1 | Root | 250 | 240695 | 56.77 | 37.41 |
| L1R | Mean ± SD |  |  | **210874 ± 26402** | **56.65** | **37.32** |
| AAU4 | L1 | Rhizospheric Soil | 250 | 626959 | 53.51 | 37.81 |
| AAU5 | L1 | Rhizospheric Soil | 250 | 617921 | 50.81 | 37.72 |
| AAU6 | L1 | Rhizospheric Soil | 250 | 671710 | 49.82 | 37.79 |
| L1RS | Mean ± SD |  |  | **638863 ±28802** | **51.38** | **37.77** |
| AU7 | L2 | Shoot | 250 | 315817 | 54.04 | 37.39 |
| AU8 | L2 | Shoot | 250 | 392595 | 56.08 | 37.34 |
| AU9 | L2 | Shoot | 250 | 248206 | 55.05 | 37.36 |
| L2S | Mean ± SD |  |  | **318873 ± 72242** | **55.05** | **37.36** |
| AU10 | L2 | Root | 250 | 230940 | 56.11 | 37.25 |
| AU11 | L2 | Root | 250 | 254326 | 55.76 | 37.62 |
| AU12 | L2 | Root | 250 | 214076 | 56.61 | 37.59 |
| L2R | Mean ± SD |  |  | **233114 ± 20212** | **56.16** | **37.48** |
| AAU10 | L2 | Rhizospheric Soil | 250 | 755028 | 49.60 | 37.95 |
| AAU11 | L2 | Rhizospheric Soil | 250 | 1102064 | 52.98 | 37.78 |
| AAU12 | L2 | Rhizospheric Soil | 250 | 779011 | 50.02 | 37.90 |
| L2RS | Mean ± SD |  |  | **878701±193809** | **50.86** | **37.87** |

**Table S2** Relative abundance of fungal taxa identified in rhizospheric soil, root and shoot of *A. mexicana* plant both locations, Amlighat and Dergaon at the phylum level

| Phylum | L1S | L1R | L1RS | L2S | L2R | L2RS |
| --- | --- | --- | --- | --- | --- | --- |
| Ascomycota | **37.47%** | **41.26%** | **75.84%** | **39.14%** | **38.06%** | **67.98%** |
| Basidiomycota | 5.52% | 3.67% | 5.35% | 3.85% | 3.53% | **6.95%** |
| Zygomycota | 0.001% | 0.002% | **5.80%** | 0.002% | 0.002% | **4.58%** |
| Unknown | 57.00% | 54.01% | 12.35% | 56.83% | 57.36% | 18.61% |
| Others | 0.009% | 0.058% | 0.66% | 0.178% | 0.048% | 1.88% |

*****Mean values of 3 replicates

**Table S3** Relative abundance of fungal taxa identified in rhizospheric soil, root and shoot of *A. mexicana* plant both locations, Amlighat and Dergaon, at the class level

| Class | L1S | L1R | L1RS | L2S | L2R | L2RS |
| --- | --- | --- | --- | --- | --- | --- |
| Saccharomycetes | **25.13%** | 10.50% | 21.84% | **20.78%** | **18.42%** | 23.13% |
| Dothideomycetes | 5.18% | 8.02% | 7.92% | 3.10% | 3.63% | 9.94% |
| Sordariomycetes | 5.79% | **27.01%** | **30.60**% | 8.75% | 15.11% | **30.13%** |
| Agaricomycetes | 4.55% | 1.19% | 4.75% | 3.34% | 2.56% | 5.70% |
| Incertae_sedis | 0.002% | 0.005% | 5.87% | 0.001% | 0.005% | 4.65% |
| Eurotiomycetes | - | 0.003% | 14.65% | - | - | 0.55% |
| Unknown | 57.31% | 51.97% | 12.74% | 62.22% | 59.51% | 20.41% |
| Others | 2.03% | 1.30% | 1.63% | 1.81% | 1.03% | 5.49% |

**Table S4** Relative abundance of fungal taxa identified in rhizospheric soil, root and shoot of *A. mexicana* plant both locations at the order level

| Order | L1S | L1R | L1RS | L2S | L2R | L2RS |
| --- | --- | --- | --- | --- | --- | --- |
| Saccharomycetales | **25.13%** | 10.49% | 21.84% | **20.78%** | **18.42%** | **23.13%** |
| Hypocreales | - | **11.80%** | **23.06%** | 2.13% | 4.14% | 17.13% |
| Phyllachorales | - | 5.85% | - | - | - | - |
| Incertae_sedis | - | 14.72% | - | - | 2.23% | 1.16% |
| Phyllachorales | 0.002% | - | - | 0.002% | 8.82% | **-** |
| Agaricales | 1.80% | - | 1.67% | - | 0.88% | 0.99% |
| Microascales | 5.16% | 1.84% | 6.86% | 7.62% | 4.55% | 7.74% |
| Capnodiales | 2.50% | - | 3.55% | 1.44% | 2.38% | 6.63% |
| Pleosporales | 2.64% | - | 4.84% | 1.63% | - | 2.16% |
| Polyporales | 2.74% | 0.39% | 2.65% | 2.31% | 1.46% | 2.70% |
| Sporidiobolales | - | - | - | 1.08% | - | - |
| Unknown | 57.32% | 52.17% | 12.96% | 62.23% | 56.83% | 22.93% |
| Mucorales | - | - | 5.51% | - | - | 2.07% |
| Eurotiales | - | - | 14.61% | - | - | 0.51% |
| Malasseziales | - | - | - | - | - | 1.72% |
| Sordariales | - | - | - | - | - | 1.97% |
| Others | 2.18% | 2.73% | 2.45% | 0.77% | 0.29% | 8.61% |

**Table S5** Relative abundance of fungal taxa identified in rhizospheric soil, root and shoot of *A. mexicana* plant the both locations, Amlighat and Dergaon at the family level

| Family | L1S | L1R | L1RS | L2S | L2R | L2RS |
| --- | --- | --- | --- | --- | --- | --- |
| Ganodermataceae | 2.73% | 0.39% | 2.15% | 2.25% | 1.46% | 2.62% |
| Unknown | 61.73% | 52.81% | 15.41% | 64.87% | 61.27% | 24.99% |
| Debaryomycetaceae | **25.06%** | 4.58% | **21.69%** | **20.72%** | **18.36%** | **22.99%** |
| Ceratocystidaceae | 5.15% | 1.84% | 6.83% | 7.61% | 2.56% | 7.69% |
| Mycosphaerellaceae | 2.50% | - | 2.36% | 1.44% | 2.38% | 6.63% |
| Incertae_sedis | - | 7.93% | - | 0.37% | 0.78% | 2.74% |
| Plectosphaerellaceae | 0.003% | 6.71% | - | 0.005% | - | 0.98% |
| Phyllachoraceae | - | 5.85% | - | - | 5.87% | **-** |
| Hypocreaceae | - | - | 0.55% | 1.27% | - | - |
| Nectriaceae | - | **11.73%** | **20.07%** | - | 3.63% | **14.10%** |
| Trichocomaceae | - | **-** | **14.61%** | - | **--** | 0.51% |
| Pleosporaceae | - | **-** | 3.15% | - | **-** | 1.00% |
| Cunninghamellaceae | - | **-** | 4.57% | - | **-** | 0.84% |
| Mortierellaceae | - | **-** | - | - | **-** | 2.49% |
| Other | 2.827% | 8.16% | 8.61% | 1.46% | 3.69% | 12.42% |

**Table S6** Relative abundance of fungal taxa at genus level identified in rhizospheric soil, root and shoot of *A. mexicana* plant

| Genus | L1S | L1R | L1RS | L2S | L2R | L2RS |
| --- | --- | --- | --- | --- | --- | --- |
| *Ceratocystis* | **5.15%** | 1.84% | **6.83%** | **7.61%** | **4.51%** | **7.69%** |
| *Fusarium* | - | - | **11.98%** | - | - | 3.36% |
| *Cladosporium* | 2.46% | - | 1.14% | 1.43% | 2.37% | 1.45% |
| *Meyerozyma* | **25.06%** | **10.46%** | **21.69%** | **20.72%** | **18.36%** | **17.70%** |
| *Rhizopycnis* | - | **7.92%** | - | - | 0.73% | - |
| *Colletotrichum* | - | 5.83% | - | - | 5.87% | - |
| *Gibellulopsis* | - | **6.69%** | - | - | - | - |
| *Penicillium* | - | - | **8.53%** | - | - | - |
| *Trichoderma* | 2.34% | 1.52% | 1.18% | 1.74% | 1.22% | 1.40% |
| *Aspergillus* | - | - | 1.87% | - | - | 0.44% |
| *Paecilomyces* | - | - | 1.02% | - | - | - |
| *Gibberella* | - | - | 4.67% | - | - | - |
| *Gongronella* | - | - | 3.62% | - | - | 0.63% |
| *Mucor* | - | - | 0.43% | - | - | 1.19% |
| *Leptosphaerulina* | - | - | 2.16% | - | - | - |
| *Mycosphaerellaceae* | - | - | - | - | - | 3.70% |
| *Debaryomycetaceae* | - | - | - | - | - | 5.28% |
| *Myrothecium* | - | - | - | - | - | 1.77% |
| Unknown | 62.22% | 64.70% | 21.75% | 65.79% | 65.32% | 29.17% |
| Others | 2.38% | 2.17% | 10.98% | 2.20% | 1.38% | 15.63% |

**Table S7** Relative abundance of fungal taxa at species level, identified in rhizospheric soil, root and shoot of *A. mexicana* plant

| Species | L1S | L1R | L1RS | L2S | L2R | L2RS |
| --- | --- | --- | --- | --- | --- | --- |
| *Rhizopycnis vagum* | - | 7.92% | - | - | 0.73% | - |
| *Aspergillus peyronelii* | - | - | 1.03% | - | - | 0.43% |
| *Gibberella intermedia* | - | - | 2.19% | - | - | - |
| Unknown | 92.78% | 76.17% | 59.03% | 90.37% | 87.90% | 66.70% |
| *Fusarium incarnatum* | - | - | 9.28% | - | - | 6.19% |
| *Eupenicillium shearii* | - | - | 1.18% | - | - | - |
| *Ceratocystis paradoxa* | 5.15% | 1.84% | 6.83% | 7.61% | 4.51% | 7.68% |
| *Fusarium oxysporum* | - | - | 1.76% | - | - | 1.84% |
| *Trichoderma longibrachiatum* | 2.01% | 1.11% | 1.20% | 1.22% | 1.24% | 1.23% |
| *Gongronella butleri* | - | - | 3.61% | - | - | 0.63% |
| *Leptosphaerulina trifolii* | -- | - | 2.16% | - | - | - |
| *Mortierella sarnyensis* | - | - | - | - | - | 1.67% |
| *Gibellulopsis nigrescens* | - | 6.69% | - | - | - | 0.61% |
| *Myrothecium verrucaria* | - | - | - | - | - | 1.74% |
| *Colletotrichum pisi* | - | 5.83% | - | - | 5.87% | - |
| Others | 2.07% | 1.55% | 11.73% | 2.02% | 0.99% | 11.28% |

**Supplementary Figures:**

A B

C D

**Figure S1** **-**Rarefaction curves of root and shoot samples of *A. mexicana* plant collected from Amlighat and Dergaon. Rarefaction curve A) Observed species, B) Chao1 curve, C) Shannon curve, D) Simpson curve.

A B

C D

**Figure S2 -**Rarefaction curves of rhizospheric soil samples of *A. mexicana* plant collected from L1 and L2. Rarefaction curve A) Observed species, B) Shannon curv, C) Chao1 curve, D) Simpson curve

A	B	C

**Figure S3** -OTU- Venn graph of root and shoot samples of *A. mexicana* representing similarity of fungal taxa in samples. Venn diagrams area reshowing the number of OTUs shared and unique among different samples. Where, A) Rhizospheric soil, B) Root sample and C) Shoot sample

A B**C**

**Figure S4 -** Relative abundance of fungal endophytes at Class level (OTUs): A) Shoot, B) Root, C) Rhizospheric Soil

A B

C

**Figure S5 -** Relative abundance of endophytic fungi at Order level (OTUs): A) Shoot, B) Root, C) Rhizospheric Soil

A B

C

**Figure S6 -** Relative abundance of endophytic fungi at Family level (OTUs): A) Shoot, B) Root, C) Rhizospheric Soil

A B

C

**Figure S7 –**Relative abundance of fungal endophytes associated with *A. Mexicana* at genus level (OTUs): A) Shoot, B) Root, C) Rhizospheric Soil

A B

C

**Figure S8 -**Relative abundance of fungal endophytes of *A. Mexicana* at species level (OTUs): A) Shoot, B) Root, C) Rhizospheric Soil

A B

**Figure S9 -**Principle component analysis of all six samples, including their replicates using Bray-Curtis index method. A) Root and Shoot, B) Rhizospheric Soil of *A. mexicana* plant

A B

C D

**Figure S10 -**Cluster analysis showing the similarity in fungal diversity in samples of *A. mexicana* plant collected from different region of Northeast India. Unweighted unifrac, A) root and shoot samples B) Rhizospheric soil samples and weighted unifrac approach, C) root and shoot samples D) Rhizospheric soil samples

A B

C

**Figure S11 -** The fungal network at phylum level from Amlighat and Dergaon. The neighbourhood connectivity of corresponding OTUs, node colour matches taxonomical orders. The thickness of lines is proportional to distance of fungal community in all samples. Here, A) Root, B) Shoot and C) Rhizospheric soil

A B

C

**Figure S12 -** The *A. mexicana* plant’s fungal network at genus level from Amlighat and Dergaon. The neighbourhood connectivity of corresponding OTUs, node colour matches taxonomical orders. The thickness of lines is proportional to distance of fungal community in all samples. Here, A) Root, B) Shoot and C) Rhizospheric soil

A B

C

**Figure S13 -** The endophytic fungal network of *A. mexicana* plant at species level from Amlighat and Dergaon. The neighbourhood connectivity of corresponding OTUs, node colour matches taxonomical orders. The thickness of lines is proportional to distance of fungal community in all samples. Here, A) Root, B) Shoot and C) Rhizospheric soil
